# MicroRNA-122-5p Inhibition Improves Inflammation and Oxidative Stress Damage in Dietary-Induced Non-alcoholic Fatty Liver Disease Through Targeting FOXO3

**DOI:** 10.3389/fphys.2022.803445

**Published:** 2022-02-11

**Authors:** Yiyi Hu, Xuetao Peng, Guoping Du, Zhiqiao Zhang, Yingji Zhai, Xingbo Xiong, Xiaoliang Luo

**Affiliations:** ^1^Department of Gestroenterology, Shunde Hospital of Southern Medical University, Foshan, China; ^2^Department of VIP Medical Center, Shunde Hospital of Southern Medical University, Foshan, China; ^3^Department of Infectious Diseases, Shunde Hospital of Southern Medical University, Foshan, China

**Keywords:** non-alcoholic fatty liver disease, miR-122-5p, FOXO3, inflammation, oxidative stress

## Abstract

Misregulated microRNA network has been emerging as the main regulator in non-alcoholic fatty liver disease (NAFLD). The deregulation of miR-122-5p is associated with the liver disease. However, the specific role and molecular mechanism of miR-122-5p in NAFLD remain unclear. In this study, we have reported that the high-fat diet (HFD) or palmitic acid (PA) significantly upregulated the hepatic miR-122-5p expression *in vivo* and *in vitro*. Inhibition of miR-122-5p suppressed accumulation-induced inflammation of lipids and oxidative stress damage in PA-treated L02 cells and HFD-induced fatty liver. The effect of the miR-122-5p inhibitor on NAFLD did not depend on insulin resistance-mediated PI3K/AKT/mammalian target of rapamycin (mTOR) signaling pathway but rather on the upregulation of its downstream FOXO3. Subsequently, we validated that miR-122-5p directly binds to the predicted 3′-UTR of FOXO3 to inhibit its gene expression. Conversely, silencing FOXO3 abolished the hepatic benefits of miR-122-5p inhibition to obese mice by decreasing the activity of antioxidant enzymes of superoxide dismutase (SOD). This study provides a novel finding that FOXO3 was the target gene of miR-122-5p to attenuate inflammatory response and oxidative stress damage in dietary-induced NAFLD. Our study provided evidence to reveal the physiological role of miR-122-5p in dietary-induced NAFLD.

## Introduction

Non-alcoholic fatty liver disease (NAFLD) is considered as a chronic progressive liver disorder that begins with simple hepatic steatosis and progresses to be non-alcoholic steatohepatitis, cirrhosis, and even liver cancer (Chiang et al., 2011). The liver is one of the major organs of insulin action, thus insulin resistance plays a pivotal role in the progression of NAFLD, which results in an increase in hepatic lipogenesis and subsequent accumulation of fatty acids in the liver (Ota et al., 2007; Bugianesi et al., 2010; Kitade et al., 2017). Accordingly, hepatic steatosis may induce inflammation response and oxidative stress damage thereby to promote the pathogenesis of hepatic insulin resistance (Gawrieh et al., 2004; Di Nunzio et al., 2010). Thus, insulin resistance intertwines with NAFLD.

Many misregulated microRNAs (miRNAs) in the liver have been identified from patients and dietary-induced murine obesity models with severe NAFLD. Some recent studies have demonstrated a potential role of miRNAs in the pathogenesis of NAFLD and insulin resistance (Cheng et al., 2016; Nie et al., 2017; Zhu et al., 2017). Binding miRNAs to the 3′-untranslated region (3′-UTR) of target-specific mRNAs can result in translational suppression or messenger RNA (mRNA) cleavage (Shukla et al., 2011). MiR-122 is one of the most abundant liver-specific miRNAs and exists in two mature isoforms: miR-122-3p and miR-122-5p. The downregulation of miR-122 is often correlated with hepatocarcinogenesis, and miR-122 is also required for the proliferation and replication of hepatitis C virus, thus antisense strategies targeting miR-122 are thought to be the potential therapeutic approach to treat hepatocellular carcinoma, hepatitis C virus, and possibly other diseases (Filipowicz and Grosshans, 2011; Thomas and Deiters, 2013). As the most abundant miRNAs are specifically expressed in the liver, miR-122-5p has been widely reported to participate in the regulation of lipid and cholesterol metabolisms, as well as the proliferation and differentiation of hepatocytes (Raitoharju et al., 2016). Recent studies have suggested that obese patients with NAFLD exhibited a higher miR-122-5p expression in the liver (Akuta et al., 2016; Latorre et al., 2017). Another study has also suggested that the serum level of circulating miR-122-5p is induced in high-fat diet (HFD)-induced NAFLD rats (Yamada et al., 2015). Our previous study has indicated that silencing miR-122-5p alleviates lipid accumulation and inflammation in L02 cells induced by oleic acid through inhibiting the TLR4/MyD88/NF-κB p65 signaling pathway (Hu et al., 2019). However, the miR-122-5p function is not clear in NAFLD.

As a key regulator in the downstream of insulin/insulin-like growth factor 1 (IGF1) signaling pathway, the forkhead box O (FOXO) transcription factors play a vital role in regulating glucose, triglyceride, and cholesterol homeostasis (Dong, 2017). A recent study has reported that miR-122-5p functioned as a protective factor in ischemic stroke by targeting FOXO3 to regulate the HSP-70-mediated NF-κB pathway *in vivo* and *in vitro* (Guo et al., 2018). As one of the numerous potential targets of miR-122-5p, FOXO3 has been reported to be implicated in the regulation of lipid metabolism, inflammation response, and fibrosis in the development of NAFLD (Dong, 2017). Therefore, targeting FOXO3 is a potential therapeutic approach for NAFLD.

We demonstrated that miR-122-5p was an important mediator for hepatic steatosis in cultured hepatocytes and HFD-induced mouse NAFLD model. Our results further demonstrated that hepatic miR-122-5p inhibition attenuated hepatic lipids accumulation-induced inflammation and oxidative stress damage through upregulating FOXO3.

## Materials and Methods

### Reagents

Media and supplies for cell culture and palmitic acid (PA) were purchased from Invitrogen (Shanghai, China). miR-122-5p mimic, miRNA negative control mimic (NC mimic), as well as the lentivirus encoding miR-122-specific miRNA antisense inhibitors (miR-122-5p inhibitor), NC inhibitor, and FOXO3 siRNA (siFOXO3) were purchased from Thermo Fisher Scientific.

### Animal Experiments

A total of 48 male C57BL/6J mice aged 8 weeks were supplied by the Chengdu Dashuo Experimental Animal Co. Ltd. (Chengdu, Sichuan, China; certificate no. SCXK201302). All animals were maintained at a constant room temperature with a 12:12-h light-dark cycle and given free access to water and food. Of them, 36 mice were fed with HFD (SLAC Animal Laboratories, Shanghai, China) for 8 weeks, providing 16% of calories from protein, 25% from carbohydrates, and 59% from fat, and 12 mice were fed with a standard chow diet (12% of fat, 29% of protein, and 59% of total carbohydrate). After 8 weeks of feeding, four mice were sacrificed to validate the hepatic steatosis and miR-122-5p levels from the HFD and chow diets, respectively. Then, 24 mice were randomly selected from the remaining HFD-fed mice and were divided into three groups (eight mice in each group). One group was treated with 50 μg of 1 × 10^10^ IU/ml control lentivirus (HFD + NC inhibitor group), while the other group was treated with 50 μg of 1 × 10^10^ IU/ml miR-122-5p antisense lentivirus (HFD + miR-122-5p inhibitor group) by intravenous tail injection. Another set of mice were injected with 50 μg of 1 × 10^10^ IU/ml miR-122-5p antisense lentivirus and 20 μl of 1 × 10^9^ TU/ml lentivirus-enveloped siFOXO3 (HFD + miR-122-5p inhibitor + siFOXO3 group). After 4 weeks of being injected with lentivirus, mice were sacrificed to validate the infection efficiency by measuring the miR-122-5p levels in the liver. All animal procedures were performed under the approval of the Animal Policy and Welfare Committee of Southern Medical University (permit no. 2019DR00121).

### Liver Histopathology

Liver tissues were fixed in 4% paraformaldehyde for 24 h and were embedded in paraffin. The paraffin sections of the liver tissues (5 μm) were prepared and stained with hematoxylin and eosin (H&E), and the frozen liver tissue sections (8 μm) were stained with *Oil Red O* (Sigma-Aldrich). Slides were mounted and observed for histological changes under a light microscope (Olympus, Japan).

### Cell Culture

Normal human liver cell line HL-7702 (L02) was obtained from the Chinese Academy of Sciences and maintained in the RPMI-1640 medium (Thermo Fisher Scientific, Inc.) containing 10% of fetal bovine serum (FBS) (Gibco, Unted States) at 37°C in a humidified chamber with 5% of CO_2_. Cells were stimulated with the indicated doses of PA. Cell culture plates were washed with phosphate-buffered saline (PBS) and fixed in 10% of neutral formalin and then stained with 0.18% of *Oil Red O* solution (Sigma-Aldrich) for 30 min. For the cell culture study, the experiments were performed in duplicate and repeated three times. The intracellular triglyceride (TG) and total cholesterol (TC) levels in L02 cells were measured and normalized to their protein contents using commercial assay kits (Applygen Technologies Inc., China), according to the instructions of the manufacturer.

### MicroRNA and Small Interfering RNA Transfection

The miR-122-5p inhibitor with its antisense or nonsense control lentiviral plasmid was constructed using a synthetic oligonucleotide containing miR-122-5p binding sites or nonsense sequence. The L02 cells were co-transfected with corresponding lentiviral vector constructs and lentiviral mix (miR-122-5p inhibitor and control lentivirus, Thermo Scientific). After 72 h of transfection, the medium was collected and concentrated using Polyethylene glycol 6000 (Sigma) precipitation. The titer was determined using the frequency of the GFP-positive L02 cells.

### Luciferase Reporter Assay

The sequence segments with wild type (WT) and mutant (Mut) seed region of FOXO3 were cloned using pGL3-Promoter Vector luciferase plasmid (Promega, Madison, WI, United States) between the *Xho*I and *Not*I sites. The L02 cells were transfected with 0.16 μg of a FOXO3 3′-UTR vector (WT and Mut) and the empty vector as well as 50 nmol/l of miR-122-5p mimic and NC mimic. After 48 h of transfection, the luciferase activity was measured using the Promega Dual-Luciferase system, and the relative luciferase activity was calculated as the ratio of firefly luciferase activity to Renilla luciferase activity.

### Real-Time Quantitative PCR

The total RNA was extracted from homogenized liver tissues and L02 cells using TRIZOL (Invitrogen, Carlsbad, CA, United States). Both reverse transcription and quantitative PCR (RT-qPCR) were performed using the Invitrogen kits (Invitrogen, Shanghai, China) on the ABI Prism 7900 Sequence Detection System (Applied Biosystems, Alameda, CA, United States). Primers for tumor necrosis factor alpha (TNF-α), interleukin (IL)-6, IL-8, and FOXO3 were synthesized using Invitrogen (Invitrogen, Shanghai, China). For miR-122-5p expression analysis, TaqMan-based miRNA RT-PCR was used. The primer sequences used are listed in **Table 1**. The mRNA and miRNA abundance were calculated using the 2^–ΔΔCt^ method and normalized to β-actin and U6, respectively.

### Enzyme Immunoassays

The cell culture medium and liver tissues were collected for determining the concentrations of TNF-α and IL-1β. The cell culture supernatants with different treatments were collected by centrifugation at 4,500 *g* for 5 min. The liver tissues were homogenized and centrifuged. Then, the supernatant was collected. The cytokine levels of TNF-α, IL-6, and IL-8 from the supernatants and liver tissues were determined using the corresponding human cytokine enzyme-linked immunosorbent assay (ELISA) kits (BioLegend, San Diego, CA, United States). The cytokine level determination was conducted for three times, and the average values were calculated. All assays were performed according to the protocols of the manufacturer.

### Western Immunoblot

Livers and cells were lysed in radio-immunoprecipitation assay (RIPA) buffer containing protease and phosphorylase inhibitors for 30 min at 4°C. Protein concentrations in the supernatant were measured using the BCA-100 Protein Quantitative Analysis kit (Biocolors, Shanghai, China). The 10 μg protein samples were separated on 10% Sodium salt-polyacrylamide gel electrophoresis (SDS-PAGE) and transferred onto PVDF membranes (Millipore). The PVDF membranes were incubated with blocking buffer [5% of non-fat dry milk and 0.1% Tween20 in TBS with Tween-20 (TBST)] for 1 h at room temperature, followed by the incubation with diluted primary antibodies overnight at 4°C with gentle shaking. The anti-FOXO3 antibody, anti-PI3K antibody, anti-AKT antibody, and anti-p-AKT antibody were obtained from Cell Signaling Technology (Beverly, MA, United States). The anti-mammalian target of rapamycin (mTOR) antibody, anti-p-mTOR antibody, and anti-β-actin were bought from Santa Cruz Biotechnology, Inc. (Dallas, TX, United States). The PVDF membrane was rinsed and incubated with horseradish peroxidase (HRP)-conjugated secondary antibodies (1:1,000, Santa) for 1 h. The protein bands were visualized by enhanced chemiluminescence detection reagents (Millipore Corporation, MA, United States) and analyzed using Image-ProPlus software (Media Cybernetics, Inc., MD, United States).

### Statistical Analysis

All data were summarized as mean ± SD from at least three independent replicates. Statistical analysis was performed with one-way ANOVA followed by a least-significant difference (LSD) *post hoc* analysis or independent samples *t*-test using SPSS version 20.0 (SPSS Inc., Chicago, IL, United States). The value *P* < 0.05 was considered as statistically significant.

## Results

### High-Fat Diet or PA Promotes miR-122-5p Expression *in vivo* and *in vitro*

**FIGURE 1:**
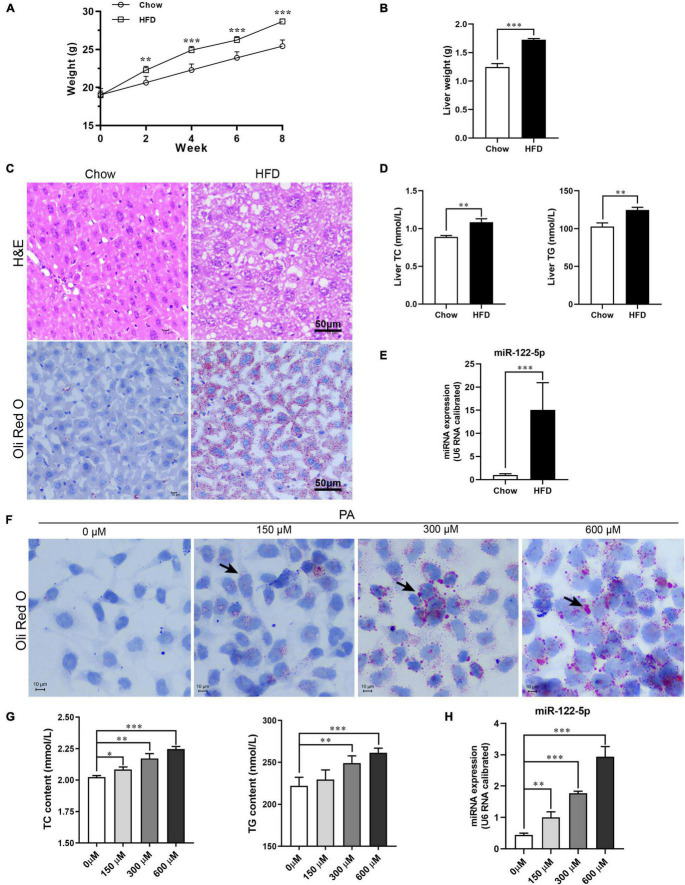
High-fat diet (HFD) or palmitic acid (PA) promotes lipid accumulation and miR-122-5p expression *in vivo* and *in vitro*. **(A)** Monitor body weight of chow-fed and HFD-fed mice. **(B)** Liver weight. **(C)** Liver morphological characteristics were observed by H&E and *Oli Red O* staining of HFD-fed mice and chow-fed mice. **(D)** Hepatic total cholesterol (TC) and triglyceride (TG). **(E)** The expression level of miR-122-5p in liver. **(F)** Representative photomicrographs of PA (0, 150, 300, and 600 μm)-induced lipid accumulation by *Oil Red O* staining. **(G)** The contents of intracellular TC and TG after treated with PA. **(H)** The miR-122-5p expression in L02 after treated with PA. Error bars indicate SD. *P < 0.05, ^**^P < 0.01, ^***^P < 0.001.

Previous studies have implied that obese patients with NAFLD presented a significantly higher level of miR-122-5p in the liver (Akuta et al., 2016; Latorre et al., 2017), so we first sought to determine whether HFD increased hepatic miR-122-5p levels in mice. After 8 weeks, HFD-fed C57BL/6J mice were notably overweight (Figure 1A), and the liver weight was significantly heavier than that in chow-fed mice (Figure 1B).

Initially, in HFD-fed mice, the hepatic adipose degeneration and lipid accumulation were observed (Figure 1C), and the levels of TC and TG in the liver were also found to be significantly increased (Figure 1D), which suggest that HFD induced hepatic steatosis of mice. The HFD significantly increased hepatic miR-122-5p levels of mice compared with standard chow-fed mice (Figure 1E). Furthermore, PA, as a toxic lipid to induce lipid accumulation of hepatic cells (Figure 1F), not only promoted the secretion of TC and TG (Figure 1G) but also upregulated miR-122-5p expression dose dependently in cultured L02 cells (Figure 1H). These data suggested that miR-122-5p expression may be correlated with the pathogenesis of NAFLD.

### Inhibition of miR-122-5p Attenuates PA-Induced Inflammatory Response and Oxidative Stress in L02 Cells

**FIGURE 2:**
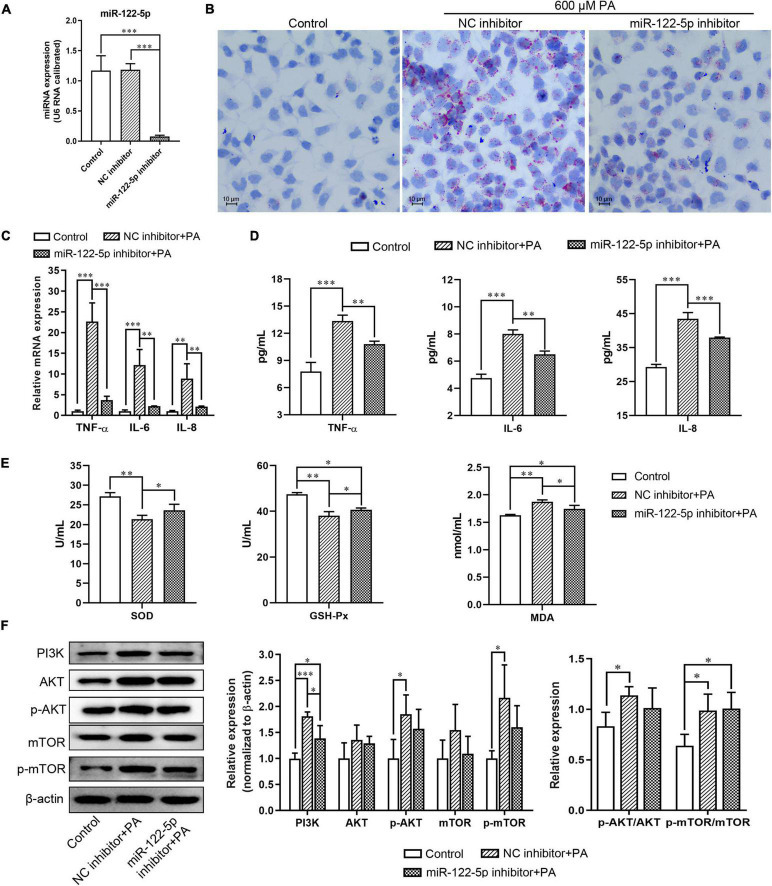
MiR-122-5p inhibition attenuates PA-induced inflammatory and oxidative stress response in L02 cells. **(A)** L02 cells were transfected with lentivirus encoding miR-122-5p antisense (miR-122-5p inhibitor) or control sense [negative control (NC) inhibitor] for 48 h, and reverse transcription and quantitative PCR (RT-qPCR) analyzed the expression level of miR-122-5p. **(B)** Representative *Oil Red O* staining. **(C)** RT-qPCR analyzed the expression of tumor necrosis factor alpha (TNF-α), interleukin (IL)-6, and IL-8. **(D)** ELISA analyzed the level of TNF-α, IL-6, and IL-8 in culture supernatants. **(E)** The activity of SOD and GSH-Px, and the content of MDA. **(F)** Western blot analysis of PI3K, AKT, p-AKT, mTOR, and p-mTOR expression. Error bars indicate SD. *P < 0.05, **P < 0.01, ***P < 0.001.

To explore the biological role of miR-122-5p in NAFLD development, L02 cells were transfected with miR-122-5p inhibitor or NC inhibitor prior to PA to silence the hepatic miR-122-5p levels (Figure 2A). MiR-122-5p inhibition significantly decreased PA-induced lipid accumulation (Figure 2B). Lipid overload of hepatocytes will cause obvious inflammatory response and inflammatory cytokine secretion (Di Nunzio et al., 2010). PA significantly upregulated the mRNA expression and secretion levels of TNF-α, IL-6, and IL-8 in L02 cells, but miR-122-5p suppression could effectively decrease their expression and secretion (Figures 2C,D).

Oxidative stress is a potential factor to promote hepatic necroinflammation and fibrosis in NAFLD development (Gawrieh et al., 2004). We found that miR-122-5p inhibitor significantly increased the levels of superoxide dismutase (SOD) and glutathione peroxidase (GSH-Px) and decreased the content of malondialdehyde (MDA) in L02 cells compared with the treatment with NC inhibitor under PA stimulation (Figure 2E). PI3K/AKT signaling plays a critical role in mediating inflammatory response in hepatocytes (Matsuda et al., 2013). As a major kinase in the insulin signaling downstream, AKT promotes hepatic lipid and protein synthesis through activating mTOR (Vander Haar et al., 2007). Furthermore, we found that PA induced L02 cells to exhibit higher expression levels of PI3K, active phosphorylated-AKT (p-AKT), and active phosphorylated-mTOR (p-mTOR) with higher ratios of p-AKT/AKT and p-mTOR/mTOR than the control group, despite the miR-122-5p inhibition attenuated PI3K expression (Figure 2F). Therefore, the miR-122-5p inhibitor could improve PA-induced inflammatory response and oxidative stress, but it might not depend on the PI3K/AKT pathway.

### Inhibition of miR-122-5p Improves HFD-Induced Oxidative Stress Injury in Fatty Liver Disease

**FIGURE 3:**
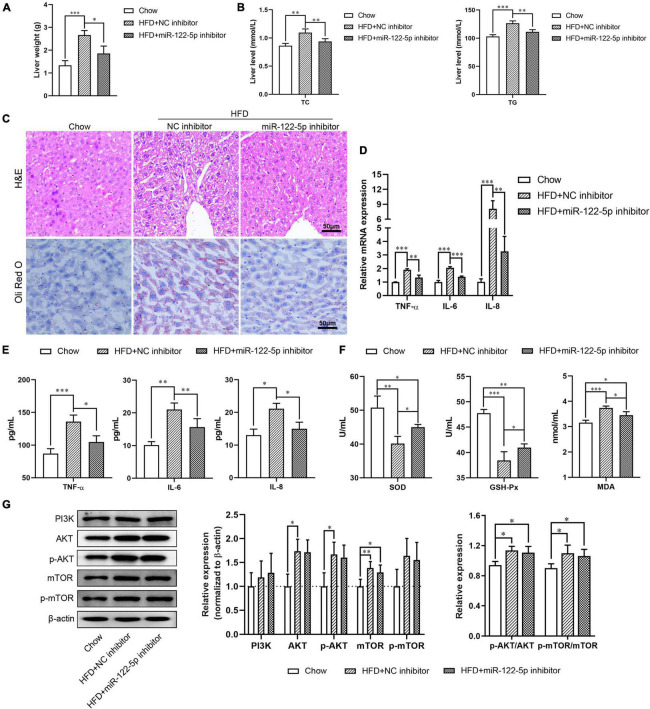
Suppression of miR-122-5p improves HFD-induced oxidative stress injury in fatty liver disease. **(A)** HFD-fed mice were injected with lentivirus encoding miR-122-5p antisense (miR-122-5p inhibitor) or nonsense (NC inhibitor) for 4 weeks, then the liver weight was measured. **(B)** Hepatic TC and TG. **(C)** Liver morphological characteristics were observed by H&E and *Oli Red O* staining after injected with miR-122-5p or NC inhibitor. **(D)** RT-qPCR analyzed the expression of TNF-α, IL-6, and IL-8. **(E)** ELISA analyzed the level of TNF-α, IL-6, and IL-8 in liver tissue. **(F)** The activity of SOD and GSH-Px, and the content of MDA. **(G)** Western blot analysis of PI3K, AKT, p-AKT, mTOR, and p-mTOR expression. Error bars indicate SD. *P < 0.05, ^**^P < 0.01, ^***^P < 0.001.

To investigate the physiological role of miR-122-5p in HFD-induced fatty liver injury, HFD-fed C57BL/6J mice aged 8 weeks were tail vein injected with miR-122-5p or NC inhibitor for another 4 weeks. As shown in Supplementary Figures 1A,B, the miR-122-5p inhibitor significantly decreased the body weight and hepatic miR-122-5p level in HFD-fed mice (*P* < 0.001). Previous studies have demonstrated that HFD-fed mice presented higher lipid deposits in hepatic tissues (Haga et al., 2015). The miR-122-5p inhibitor also reduced liver weight as well as the levels of TC and TG in the liver of HFD-fed mice as compared with the NC inhibitor group (Figures 3A,B). In histological analysis, we found that miR-122-5p inhibitor could effectively ameliorate the histological feature of hepatic steatosis, which mainly exhibited excessive lipid accumulation and immune cell infiltration (Figure 3C). Furthermore, miR-122-5p suppression also downregulated the mRNA expression and secretion levels of inflammatory cytokines (TNF-α, IL-6, and IL-8) (Figures 3D,E) and improved liver antioxidative stress capacity by increasing the activities of SOD and GSH-Px and decreasing the content of MDA (Figure 3F). HFD activated the PI3K/AKT/mTOR signaling pathway in the liver, but the miR-122-5p silence could not effectively inactivate the PI3K/AKT/mTOR signaling pathway *in vivo* (Figure 3G). Collectively, these data suggested that suppression of miR-122-5p played a protective role in oxidative stress injury *in vitro* and *in vivo*.

### MiR-122-5p Directly Inhibits FOXO3 Expression in L02 Cells and Hepatic Tissues in Obese Mice

**FIGURE 4:**
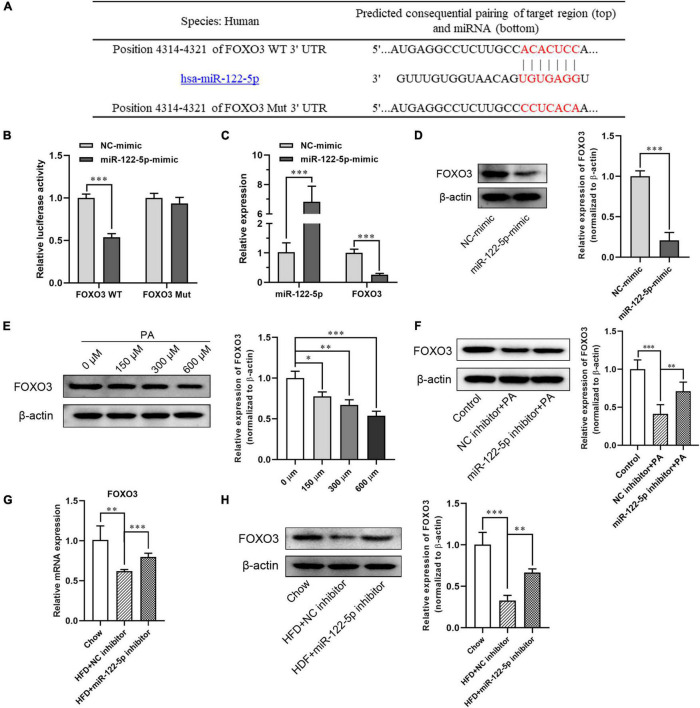
MiR-122-5p directly inhibits FOXO3 expression in L02 cells and hepatic tissues in obese mice. **(A)** Matched sequence (red box) of with mature miR-122-5p and the FOXO3 WT. **(B)** Targeted binding of miR-22-5p and FOXO3 were detected by fluorescein reporter. **(C)** qRT-PCR analysis of miR-122-5p and FOXO3 expression in L02 cells after transfection with miR-122-5p mimic. **(D)** Western blot analysis of FOXO3 expression after transfection with miR-122-5p mimic. **(E)** Western blot analysis of FOXO3 expression in L02 cells treated with different concentrations of PA. **(F)** Western blot analysis of FOXO3 expression in L02 cells treated with miR-122-5p inhibitor. **(G)** qRT-PCR analysis of hepatic FOXO3 expression after injected with miR-122-5p inhibitor. **(H)** Western blot analysis of hepatic FOXO3 expression after injected with miR-122-5p inhibitor. Error bars indicate SD. *P < 0.05, ^**^P < 0.01, ^***^P < 0.001.

To further investigate the molecular mechanism of miR-122-5p protecting against inflammatory and oxidative stress damage in fatty liver disease, the miRNA target analysis tools (miRBase, TargetScan, and mirecords.biolead.org/) were used for the prediction of the potential targets of miR-122-5p. In numerous potential targets, FOXO3 was selected for further analysis as it has been reported to be closely associated with lipid metabolism and insulin resistance in HFD-induced NAFLD (Zhu et al., 2017). As shown in Figure 4A, as FOXO3 3′-UTR contains miR-122-5p binding sequence, we constructed luciferase reporters encoding FOXO3 WT and Mut 3′-UTR then to be co-transfected with plasmid encoding miR-122-5p into L02 cells. Through luciferase reporter assay, we found that miR-122-5p could directly bind to FOXO3 3′-UTR but not to mutant FOXO3 3′-UTR. This result demonstrated that FOXO3 was a potential target of miR-122-5p (Figure 4B). In addition, we noted that miR-122-5p overexpression significantly inhibited the mRNA and protein expression of FOXO3 in L02 cells (Figures 4C,D).

Later, we analyzed the inhibition of miR-122-5p on FOXO3 expression in PA-treated L02 cells and hepatic tissues in HFD-induced NAFLD mice. In contrast to the increment of miR-122-5p expression in cultured L02 cells, FOXO3 protein expression was downregulated dose dependently after PA treatment (Figure 4E). While miR-122-5p inhibitor could significantly increase FOXO3 expression of PA-treated L02 cells *in vitro* (Figure 4F). Furthermore, suppression of miR-122-5p also significantly promoted FOXO3 mRNA and protein expression in HFD-induced NAFLD mice *in vivo* (Figures 4G,H). Collectively, these data suggested that miR-122-5p inhibited FOXO3 expression *in vitro* and *in vivo* by binding to its 3′-UTR region.

### MiR-122 Inhibited HFD-Induced Fatty Liver Disease by Targeting FOXO3

**FIGURE 5:**
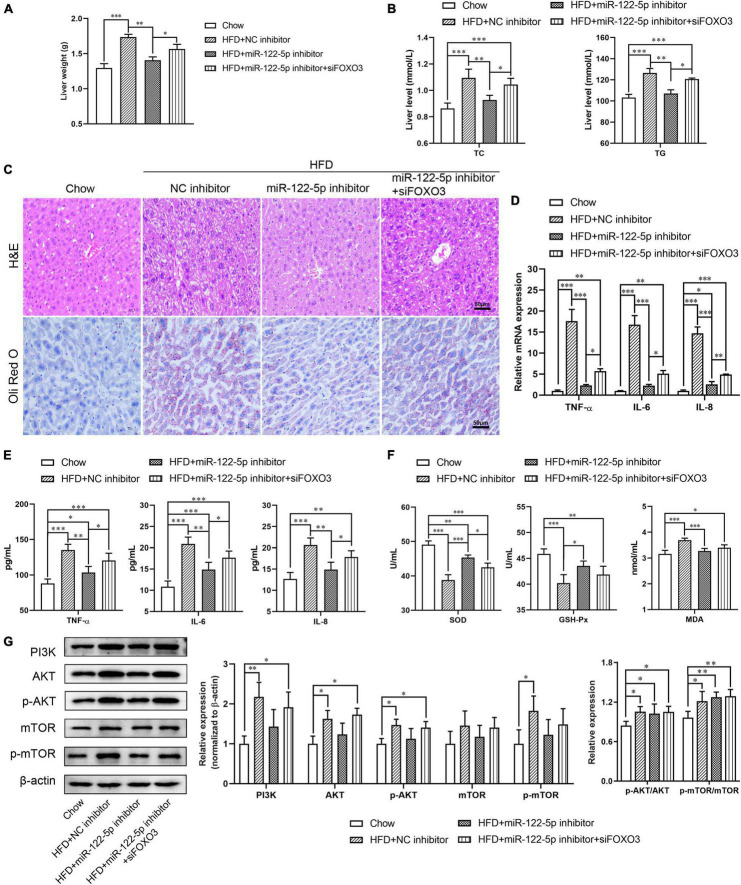
Silence FOXO3 abolishes the benefits of miR-122-5p inhibition in hepatic tissue. **(A)** Liver weight. **(B)** Hepatic TC and TG. **(C)** H&E and *Oli Red O* staining for liver morphological characteristics. **(D)** RT-qPCR analyzed the expression of TNF-α, IL-6, and IL-8. **(E)** ELISA analyzed the level of TNF-α, IL-6, and IL-8 in liver tissue. **(F)** The activity of SOD and GSH-Px, and the content of MDA. **(G)** Western blot analysis of PI3K, AKT, p-AKT, mTOR, and p-mTOR expression. Error bars indicate SD. *P < 0.05, ^**^P < 0.01, ^***^P < 0.001.

To further explore the impact of miR-122 on the function of FOXO3 in HFD-induced liver injury, we used FOXO3 siRNA to figure out whether blocking FOXO3 could reverse the benefits of miR-122-5p inhibition. As shown in Supplementary Figures 1C,D, FOXO3 siRNA treatment downregulated hepatic FOXO3 expression at mRNA and protein levels in miR-122-5p inhibitor-injected obese mice. Compared with the benefits of miR-122-5p inhibitor-treated obese mice, si-FOXO3 reversely increased liver weight and TC and TG levels (Figures 5A,B) and aggravated lipid accumulation and immune cell infiltration (Figure 5C). Furthermore, siFOXO3 exhibited higher inflammatory cytokine expression and lower antioxidative stress levels than miR-122-5p inhibitor-treated mice (Figures 5D–F). Since reactivation of FOXOs might protect against hepatic fibrosis through attenuating cell proliferation and transdifferentiation of hepatic stellate cells, FOXOs dysregulation may be implicated in NAFLD development (Dong, 2017). PI3K/AKT signaling leads to nuclear exclusion of phosphorylated FOXOs and consequently reduces the FOXOs transcriptional activity for hepatic gluconeogenesis (Webb and Brunet, 2014). In other words, FOXO3 is the immediate downstream effectors of PI3K/AKT signaling. Interestingly, neither inhibition of miR-122 nor FOXO3 silence could activate the PI3K/AKT signaling pathway and mTOR (Figure 5G). Those results demonstrated that the hepatic protective effect of miR-122-5p inhibition was dependent on the upregulation of FOXO3.

## Discussion

In recent years, the role of miRNA-mediated gene regulation in liver function and diseases has attracted more attention. Misregulated miRNAs has been reported to be involved in NAFLD pathogenesis, such as hepatic inflammatory response, oxidative stress damage, and lipid accumulation (Castro et al., 2013; Yamada et al., 2013; Torres et al., 2018). In this study, we provided novel evidence that miR-122-5p hepatic silencing abrogated HFD-induced liver lipid accumulation, inflammation response, and oxidative stress damage through directly upregulating the transcriptional factor FOXO3.

MiR-122-5p is highly expressed in liver tissues, especially in hepatocytes, and positively related to fatty liver and related lipoprotein metabolism (Raitoharju et al., 2016). Previous studies have implied that obese patients with NAFLD exhibited a significantly higher level of miR-122-5p in the liver (Akuta et al., 2016; Latorre et al., 2017). However, no evidence has directly unveiled the physiological function of miR-122-5p in dietary-induced NAFLD. In this study, we found that HFD feeding increased miR-122-5p level in the liver of mice and overload of PA stimulated miR-122-5p expression in hepatocytes. Excess energy uptake during HFD-induced obesity leads to ectopic lipid accumulation, which is highly ascribed to deposition of lipids in non-adipose tissues, including skeletal muscle or liver (Farrell and Larter, 2006; Tilg and Moschen, 2010). With lipid overload in hepatocytes, toxic lipids trigger pro-inflammatory signaling and activate transcriptional factors of NF-κB and AP-1 to induce the secretion of inflammatory cytokines, such as TNF-α, IL-6, and IL-8, which are considered to be the major pro-inflammatory mediators in NAFLD (Joshi-Barve et al., 2007; Di Nunzio et al., 2010; Pal et al., 2012). Moreover, the reduced antioxidant level accelerates NAFLD by inducing lipid peroxidation and promoting insulin resistance and inflammation response (Ji et al., 2019). Consistent with our previous study in oleic acid-treated L02 cells (Hu et al., 2019), we found that hepatic miR-122-5p inhibition attenuated lipid accumulation and the expression of inflammatory cytokines in PA-treated L02 cell and HFD-induced fatty liver. Furthermore, this study also indicated that miR-122-5p inhibition improved the activities of antioxidant enzyme SOD and GSH-Px to decrease lipid peroxidation and the generation of MDA. Although the mechanism behind the regulation of miR-122-5p production is still unclear, miR-122-5p production may promote the lipid accumulation, inflammation response, and oxidative stress damage of the liver to HFD.

Insulin resistance is a driver of hepatic steatosis progression, and oxidative stress induced by lipid peroxidation may be a potential factor promoting hepatic gangrenous inflammation and fibrosis (Gawrieh et al., 2004). Insulin/IGF1 activates tyrosine kinase activity through insulin receptor/IGF1 receptor to induce insulin receptor substrate phosphorylation, which subsequently stimulates phosphoinositide 3-kinase (PI3K) to activate AKT (Arden, 2008; Webb and Brunet, 2014). As a major downstream kinase of the insulin signaling, AKT promotes hepatic lipid and protein synthesis through activating mTOR (Vander Haar et al., 2007). However, we found that there was no difference in the hepatocellular PI3K/AKT pathway between NC inhibitor group and miR-122-5p inhibitor group *in vivo* and *in vitro*. Therefore, it was suggested that the improvements in miR-122-5p inhibitor on PA-induced or HFD-induced inflammatory response and oxidative stress might not depend on the PI3K/AKT pathway.

As a key downstream regulator in the insulin/IGF1 signaling pathway, FOXO transcription factors have been involved in numerous cellular functions, including the mediation of glucose, triglyceride, and cholesterol homeostasis (Webb and Brunet, 2014; Dong, 2017). In this study, we identified that miR-122-5p directly targeted hepatic FOXO3 and inhibited its gene expression *via* dual-luciferase assay. Therefore, miR-122-5p inhibition could downregulate FOXO3 expression *in vitro* and *in vivo*. Recently, Wang et al. (2019) reported that FOXO3 promotes the transcriptional activity of the SREBP1c promoter, thus leading to increased TG synthesis and hepatic TG accumulation in the high glucose and high PA-induced HepG2 cells and the obese mice under insulin resistance. Upon insulin stimulation, the PI3K/AKT signaling pathway is activated by the insulin signaling cascade (Cai et al., 2017), which will inhibit the FOXO3 transcriptional activity by phosphorylation (Dong, 2017; Manning and Toker, 2017). Therefore, under the physiological conditions of FOXO3 inactivation induced by insulin-dependent phosphorylation, this FOXO3-SREBP1c pathway-mediated lipid synthesis would be inhibited. Interestingly, in the PA-induced L02 cells and HFD-fed mice, we found that FOXO3 expression was downregulated under the activation of PI3K/AKT signaling pathway. These results indicated that the HFD-induced NAFLD mice and PA-induced L02 cells did not develop an insulin-resistant state in our study, since the insulin sensitivity varies among various cell types and animals.

Notably, FOXO1/3 or FOXO1/3/4 genes knockout in mouse liver, respectively, resulted in a mild or moderate hepatic steatosis on a standard chow diet (Tao et al., 2011; Pan et al., 2017). When challenged with HFD-specific FOXO1/3/4, knockout mice have a phenotype with very severe hepatic steatosis, as well as severe inflammation and fibrosis, especially in response to a high-fat plus cholesterol diet (Pan et al., 2017). Transgenic overexpression of a constitutively active FOXO3 decreases hepatic triglyceride level, indicating that FOXO3 could reverse hepatic steatosis (Pan et al., 2017). Therefore, FOXO3 may have opposite regulatory effects on hepatic steatosis under insulin-dependent and non-independent signaling. Our further study determined that the benefits of miR-122-5p inhibitor in obese mice could be abolished by silencing FOXO3, including decreasing lipid accumulation, which also demonstrated that miR-122-5p targeting on FOXO3 played a crucial role in obesity-induced mouse fatty liver diseases. Nevertheless, the contribution of other miR-122-5p target genes to lipogenesis cannot be ruled out, and the gain- and loss-of-function analyses of miR-122-5p *in vivo* and *in vitro* suggested that miR-122-5p was able to promote NAFLD development through inhibiting FOXO3 function. However, further studies are needed to clarify whether the role of miR-122-5p in lipid synthesis modulation is related to targeting FOXO3.

Oxidative stress is a key induction factor in promoting the lesion development of fatty liver to non-alcoholic steatohepatitis (Friedman et al., 2018). The imbalance of reactive oxygen species (ROS) and antioxidant molecules in the liver produces oxidative stress, which can lead to lipid peroxidation and the production of inflammatory cytokines, and even contribute to hepatocellular injury and fibrosis, promoting the progression from simple steatosis to non-alcoholic steatohepatitis (Peverill et al., 2014). Therefore, an antioxidative therapeutic strategy holds the potential to treat liver steatosis and inflammation. FOXO3 is not only a direct transcriptional regulator for gluconeogenesis but is also required for cellular antioxidant defense in liver diseases. FOXO3 reduces cellular ROS levels through upregulating the antioxidant enzymes CAT and SOD, which contributes to preserving the mitochondrial reserve capacity and thus protect hepatic cells from oxidative damage (Marinkovic et al., 2007; Davila and Torres-Aleman, 2008). In this study, we found that miR-122-5p inhibition upregulated FOXO3 expression and improved the levels of antioxidant enzymes SOD and GSH-Px, as well as decreased the generations of MDA and inflammatory cytokines. However, silencing FOXO3 decreased its downstream SOD activity and increased the levels of lipid peroxidation product MDA and inflammatory cytokines in miR-122-5p inhibitor-treated NAFLD mice, indicating that miR-122-5p may be an important negative regulator for the FOXO3-mediated antioxidant stress signaling pathway. It is reported that reactivation of FOXO3 can protect against hepatic fibrosis through attenuating cell proliferation and transdifferentiation of hepatic stellate cells (Dong, 2017). Mice that are deficient in hepatic FOXO3 are more susceptible to non-alcoholic steatohepatitis than WT controls (Pan et al., 2017), which provided strong evidence for FOXO3 protecting against the diet-induced fatty liver disease. Our findings supported that miR-122-5p could promote HFD-induced NAFDL *via* inhibiting FOXO3-mediated antioxidative stress pathway to aggravate the inflammatory response. It revealed that the genetic or pharmaceutical modification of miR-122-5p was a promising method to counteract obesity and related metabolic diseases.

Collectively, our results demonstrated that miR-122-5p was a potential mediator to contribute to hepatic inflammatory and oxidative stress response through inhibiting FOXO3 in the development of NAFLD. Therefore, miR-122-5p/FOXO3 might be a potential therapeutic agent against obesity-related NAFLD.

## Data Availability Statement

The original contributions presented in the study are included in the article/Supplementary Material, further inquiries can be directed to the corresponding author.

## Ethics Statement

The animal study was reviewed and approved by all animal procedures were approved by the Animal Policy and Welfare Committee of Southern Medical University with a permit number (2019DR00121).

## Author Contributions

YH and GD conceived and designed the experiments. YZ, ZZ, and XL performed the experiments. YH and XP analyzed the data and contributed to the reagents and materials. YH and GD wrote the manuscript. All authors contributed to the article and approved the submitted version.

## Conflict of Interest

The authors declare that the research was conducted in the absence of any commercial or financial relationships that could be construed as a potential conflict of interest.

## Publisher’s Note

All claims expressed in this article are solely those of the authors and do not necessarily represent those of their affiliated organizations, or those of the publisher, the editors and the reviewers. Any product that may be evaluated in this article, or claim that may be made by its manufacturer, is not guaranteed or endorsed by the publisher.
