# Supplementary material for: MicroRNA-122-5p Inhibition Improves Inflammation and Oxidative Stress Damage in Dietary-Induced Non-alcoholic Fatty Liver Disease Through Targeting FOXO3
Source: Front Physiol. 2022 Feb 11;13:803445. doi: 10.3389/fphys.2022.803445 (PMC8874326; doi:10.3389/fphys.2022.803445)
Supplement: Supplementary file 1 [file Image_1.pdf]

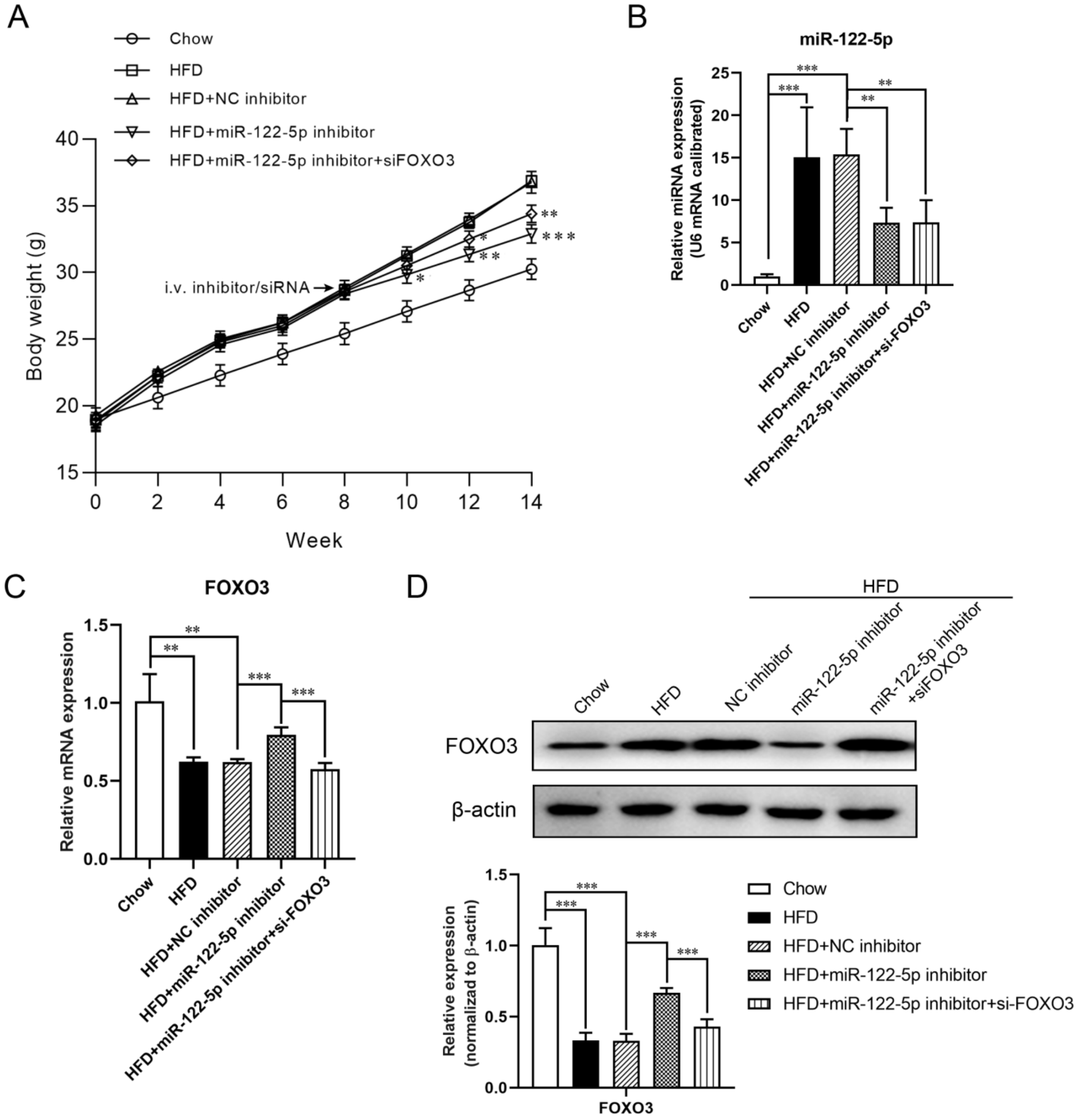

Supplementary Fig. 1 Effect of miR-122-5p inhibitor and FOXO3 siRNA on body weight and the expression of hepatic miR-122-5p and FOXO3 in HFD-induced obese mice. (a) Body weight. (b) qRT-PCR analysis of hepatic miR-12-5p expression. (c) qRT-PCR analysis of hepatic FOXO3 expression. (d) Western blot analysis of hepatic FOXO3 expression. Error bars indicate SD. **P*<0.05, ***P*<0.01, ****P*<0.001.
